# miR-185-5p response to usnic acid suppresses proliferation and regulating apoptosis in breast cancer cell by targeting Bcl2

**DOI:** 10.1186/s40659-020-00285-4

**Published:** 2020-05-04

**Authors:** Elif Değerli, Vildan Torun, Demet Cansaran-Duman

**Affiliations:** Biotechnology Institute, Ankara University, Keçiören, 06135 Ankara, Turkey

**Keywords:** miR-185-5p, Usnic acid, Breast cancer, Apoptosis, BCL2

## Abstract

**Background:**

Breast cancer is the most common cancer types among women. Recent researches have focused on determining the efficiency of alternative molecules and miRNAs in breast cancer treatment. The aim of this study was to determine the effect of usnic acid response-miR-185-5p on proliferation in the breast cancer cell and to determine its relationship with apoptosis pathway.

**Methods:**

The cell proliferation and cell apoptosis rate were significantly increased following the ectopic expression of miR-185-5p in BT-474 cells. Furthermore, the results of cell cycle assay performed by flow cytometry revealed that the transfection with miR-185-5p induced G1/S phase arrest. The apoptosis-related genes expression analysis was performed by qRT-PCR and the direct target of miR-185-5p in BT-474 cells was identified by western blot and luciferase reporter assay.

**Results:**

Our data showed that miR-185-5p can cause significant changes in apoptosis-related genes expression levels, suggesting that cell proliferation was suppressed by miR-185-5p via inducing apoptosis in breast cancer cells. According to western blot results, miR-185-5p lead to decrease BCL2 protein level in BT-474 cells and direct target of miR-185-5p was identified as BCL by luciferase reporter assay.

**Conclusion:**

This study revealed that miR-185-5p may be an effective agent in the treatment of breast cancer.

## Background

Breast cancer is the most common cancer among women and morbidity of this disease increases every year in the world. Breast cancer is a heterogeneous disease with different histological and molecular characteristics according to the data obtained from gene expression analysis. The surgery, chemotherapy, radiation and the combination of these therapeutic approaches are used in cancer patients for cancer management and therapy stage. Each of the therapy method has some advantages and disadvantages. Chemotherapies have been determined to be effective in the early stages of breast cancer by interfering with DNA synthesis and tumor replication. Besides, chemotherapies play a role as adjuvant therapy to reduce micrometastases and cancer recurrence or to reduce tumor as a neoadjuvant [1]. The endocrine, chemotherapeutic and biological therapies which include Trastuzumab, Pertuzumab, Lapatinib, and Neratinib are used to produce a positive effect and a reduced death rate for breast cancer patients [2]. But, the traditional non-surgical therapies are associated with significant toxicity and some individuals due to drug resistance [2, 3]. The novel molecules derived from biological origin such as lichens can kill the cancer cells by the induction of apoptosis with the minimum side effect on non-cancerous cells. Induction of apoptosis is one of the most important steps of cytotoxic anticancer agents. Novel drug molecules induce apoptosis pathways that are blocked in cancer cells by manipulating different mechanisms in cancer cells [4, 5]. Therefore, the development of novel molecular targeted therapies is required for many patients as different therapeutic options [2].

Natural compounds derived from plants, fungi, and lichens have long been a source of anticancer compounds for cancer treatments for many years. The valuable compounds obtained from biological organisms are generally low in cost, plentiful in nature, and cause little toxicity or side effects in applications. Usnic acid is well-known as medicinal lichen secondary metabolite and is a potentially interesting candidate to use in cancer treatment [6]. The activity of usnic acid has been investigated in various pathways including cytotoxicity, regulation of the cell cycle, stimulation of cell death, modulating mechanisms of immune activity, angiogenesis and energy metabolism [7]. Usnic acid can inhibit tumor cell growth, metastasis, and proliferation, and induce the apoptosis of different cancer cells through a signaling cascade of death receptor-mediated extrinsic and mitochondria-mediated intrinsic caspase pathways [8, 9]. Besides, usnic acid has a high anti-proliferative effect against cancer cells, whereas it has little effect on normal epithelial cells [6, 10]. This indicates that usnic acid may be a very important therapeutic molecule in cancer treatment.

Recent advances promise hope for cancer treatment by regulating the gene expression of non-coding small RNAs. These developments aim to provide new therapeutic approaches for cancer treatment and to expand a new method to inhibit cancer by miRNA [11, 12]. MicroRNAs (miRNAs) are endogenous, highly conserved, and small noncoding RNAs of about 22 nucleotides in length that regulate the gene expression at the posttranscriptional level [13]. Since avoiding from apoptosis is a critical characteristics of malignant tumors, miRNAs are well known to have key roles in apoptosis regulation [14, 15]. miRNAs as candidate therapeutic molecules suppress tumor formation by regulating apoptosis and this needs to be addressed [2]. Given that most chemotherapeutic drugs kill cancer cells through apoptosis and miRNAs are also involved in the regulation of apoptosis, it is obvious that miRNAs can also be an effective target for cancer therapies [2]. The number of miRNA profiling studies have rapidly increased in recent decades. Many studies have demonstrated that miRNAs play key roles in the occurrence and progression of malignancies including breast cancer [16, 17]. Moreover, several studies have revealed that the dysregulation of miRNA expression is involved in cancer, suggesting their function as either oncogene (oncomir’s) or tumor suppressor in a tissue-specific manner [18]. Pre-clinic and clinic studies include the evaluation of miRNAs as therapeutic agents in recent years. Numerous preclinical studies have revealed miR-34a as a tumor suppressor is against various types of cancers through targeting of many oncogenic pathways [19]. Phase I clinical study on the safety, pharmacokinetics, and effectiveness of a liposome-encapsulated synthetic miR-34a mimic, namely MRX34, was conducted in patients with advanced solid tumors [20, 21].

Unlike chemically synthesized drugs, the effect of usnic acid on breast cancer treatment was investigated at the molecular (miRNA) level in the previous studies [22]. Kılıç et al. investigated the anti-proliferative effect of usnic acid, an alternative drug candidate molecule, on different breast cancer cell lines (MDA-MB-231, BT-474 and MCF-7). The inhibitory effect of usnic acid on cell growth was determined in breast cancer cell lines and various miRNAs that respond to usnic acid in three different breast cancer cells were identified by miRNA microarray analysis. With bioinformatics and validation studies, the most effective miRNA associated with BT-474 cell line treated with usnic acid was determined as miR-185-5p in our previous study [21]. According to Kılıç et al. studies, the miRNA targets of usnic acid were mostly found to play role in Hedgehog signaling pathway, Transforming growth factor beta (TGF-Beta), Mitogen-activated protein kinase (MAPK) and apoptosis pathways [21]. In the light of these data, this is the first study which give comprehensive information about the changes in the expression level of miRNAs following the application of usnic acid to breast cancer cells, apoptosis pathways targeted by these miRNAs and how these events can be exploited in breast cancer cells.

In the current study, we investigated the effects of overexpression of miR-185-5p on proliferation and apoptosis in a breast cancer cell in vitro. This is the first study that the efficiency of miR-185-5p on cell apoptosis was investigated in BT-474 breast cancer cell line.

## Materials and methods

### Cell culture

Human breast cancer BT-474 cell line and human breast epithelial MCF-12A cell line obtained from ATCC. BT-474 cells were cultured in Dulbecco’s modified Eagle’s medium (DMEM) (Sigma, USA) with high glucose containing 10% Fetal Bovine Serum (FBS) (Biological Industries, Israel) and 1% penicillin/streptomycin (Biowest, USA) at incubated 37 °C, 5% CO_2_. MCF-12A cells were maintained in DMEM Ham’s F12 (Sigma, USA) including 10% FBS, 1% penicillin/streptomycin, l-glutamine, 10 μg/ml insulin, 20 ng/ml Epidermal Growth Factor (EGF) (Goquick) and 0.5 mg/ml hydrocortisone (PubChem) at 37 °C with 5% CO_2_.

### Cell viability and proliferation analysis

Cell viability of BT-474 and MCF-12A was determined by the MTT (3-(4,5-dimethylthiazol-2-yl)-2,5-diphenyltetrazolium bromide) assay (Sigma) according to the manufacturer’s protocol. 1 × 10^5^ cells per well were seeded in 96 well-plate and after 24 h, cells transfected with miRNA mimics and scramble in different concentrations of 0, 10, 15, 25 and 50 nM. After 24, 48, and 72 h incubation time, 10 µl MTT (5 mg/ml) was added to each well and the plates were incubated at 37 °C for 4 h. After 4 h incubation, dimethyl sulfoxide (DMSO) of 100 µl was added to each sample and the absorbance was measured at 570 nm using microplate reader (PerkinElmer 1420 Multilabel Counter, USA). Each experiment was performed three times.

### miRNA transfection

miRNA mimics and scramble transfection were carried out by HiPerfect Transfection Reagent (Qiagen, Germany) according to the manufacturer’s protocol. miRNA mimics and scramble were separately transfected to BT-474 and MCF-12A cells in the final concentration of 25 nM. These transfected cells were incubated for 48 h and then cells were collected for further investigations.

### The validation of transfection

miR-185-5p (5′UGGAGAGAAAGGCAGUUCCUGA3′) transfection into cells was validated by using qRT-PCR. The thermal cycling parameters used for amplification were as follows: initial activation at 95 °C for 15 min, followed by 40 cycles at 94 °C for 15 s, 55 °C for 30 s, and 70 °C for 30 s. The relative expression level of miR-185-5p was calculated by using 2^−ΔΔCt^ formula, normalizing with RNU-6 (5′CGCAAGGATGACACGCAAATTC3′) housekeeping gene.

### Cell-cycle analysis

BT-474 cells (5 × 10^5^)/well were seeded into six-well plates. After 48 h transfection, harvested cells were washed with cold PBS and fixed with 70% ethanol at − 20 °C for at least 2 h. Propidium iodide (PI)/RNase staining buffer (BD Biosciences, USA) was used to analyze cell cycle. 1 × 10^6^ cells were resuspended in 500 µl PI/RNase staining buffer and the cells were incubated 15 min at room temperature and analyzed by flow cytometry at 488 nm (BD Accuri Plus, US). The cells used in the control and experimental group were synchronized to perform cell cycle assay.

### Apoptosis detection assay

Cell apoptosis assay was performed Annexin V-FITC/PI apoptosis assay kit (Invitrogen, USA). Briefly, BT-474 and MCF-12A cells (5 × 10^5^)/well were seeded into six-well plates. After 48 h miRNA transfection process, treated cells were harvested and washed twice with cold PBS. The treated cells were resuspended with 1 µl YO-PRO and 1 µl PI and incubated in ice for 30 min. Cell apoptosis rate was measured with flow cytometry (BD Accuri Plus, US).

### Quantification of mRNA with reverse transcription polymerase chain reaction (qRT-PCR)

Total RNA extraction from transfected and non-transfected cells was performed using Genezol Reagent (Geneaid, Taiwan), in accordance with the manufacturer’s protocol. NG dART RT kit (EURx, Poland) was used to reverse transcription according to the manufacturer’s instructions. The reverse transcription reaction conditions were 10 min at 25 °C, 30 min at 48 °C, and a final step of 5 min at 95 °C. The level of gene expression was determined by Human Apoptosis Primer Library (Real Time Primers, US), containing 88 apoptosis-related genes and 8 housekeeping genes. All qRT-PCR experiments were repeated three times.

### Western blot analysis

Cultured cells in the six-well plate were harvested and washed with cold phosphate-buffered saline (PBS). The protein isolation from cell pellets was carried out using lysis buffer containing 7 M Urea, 2 M Thiourea, 4% Chaps, 1% dichlorodiphenyltrichloroethane (DDT), 2% Ampholyte and supplemented with protease inhibitor. The protein amounts for each sample were quantified using BCA™ Protein Assay Kit (Merck, Germany) and an equal amount of protein from each sample was separated by 12% sodium dodecyl sulfate-polyacrylamide gel electrophoresis (SDS-PAGE). After the separation process, proteins were transferred onto a nitrocellulose membrane (Thermo Fisher Scientific, USA), followed by blocking for 1 h with 5% nonfat milk. The membrane was incubated at 4 °C overnight with diluted anti-BCL2 primary antibodies (Santa Cruz Biotechnology, USA) and anti-β-Actin primary antibodies (FineTest, China) as a control. Subsequently, the membranes were washed with Tris-Buffered Saline Tween 20 (TBST) buffer for 3 times. After washing, the membranes were incubated with horseradish peroxide (HRP) conjugated secondary antibodies (Abcam, US) at room temperature for 1.5 h. Finally, the chemiluminescence kit (Thermo Fisher Scientific, USA) was used to visualize immunoreactive bands and evaluated with ImageJ program.

### Luciferase reporter assay

BT-474 cells (3 × 10^4^ cells/well) were seeded into a 96-well plate and cultured for 24 h. Subsequently, the cells were co-transfected with miR-185-5p (Qiagen, USA) and luciferase reporter plasmid containing 3′UTR of BCL2 (OriGene, USA) and transfected with luciferase reporter plasmid as a control group by using Lipofectamine 2000 (Thermo Fisher Scientific, USA) according to manufacturer’s protocol. After 24 h transfection, luciferase assay was performed using Luciferase Assay Systems (Promega, USA) and luciferase activity was measured by a luminometer (PerkinElmer 1420 Multilabel Counter, USA).

### miRNA target prediction

miRSystem (http://mirsystem.cgm.ntu.edu.tw/), miRDB (http://mirdb.org/), and DIANA (http://diana.imis.athena-innovation.gr/DianaTools/index.php) bioinformatics tools were used to predict in silico miRNA targets and 3′UTR BCL2 site binding with seed region was determined by miRmap (https://mirmap.ezlab.org/) bioinformatics tool.

### Statistical analysis

Data are presented as the means ± (SD) from at three separate experiments of the apoptosis, cell cycle assay, western blot, and luciferase assay analysis. Differences between experiment and control group were defined with two-tailed Student’s t-tests and one-way ANOVA. p values ˂ 0.05 were considered significant. For qRT-PCR analysis, GAPDH was used as housekeeping gene and qRT-PCR data was analyzed according to the ΔΔC_T_ method and SPSS16.0 software. Graphpad Prism 8.0 software was used to represent data on graph. 

## Results

### miR-185-5p suppresses proliferation of BT-474 cells in a dose-dependent manner

**Fig. 1:**
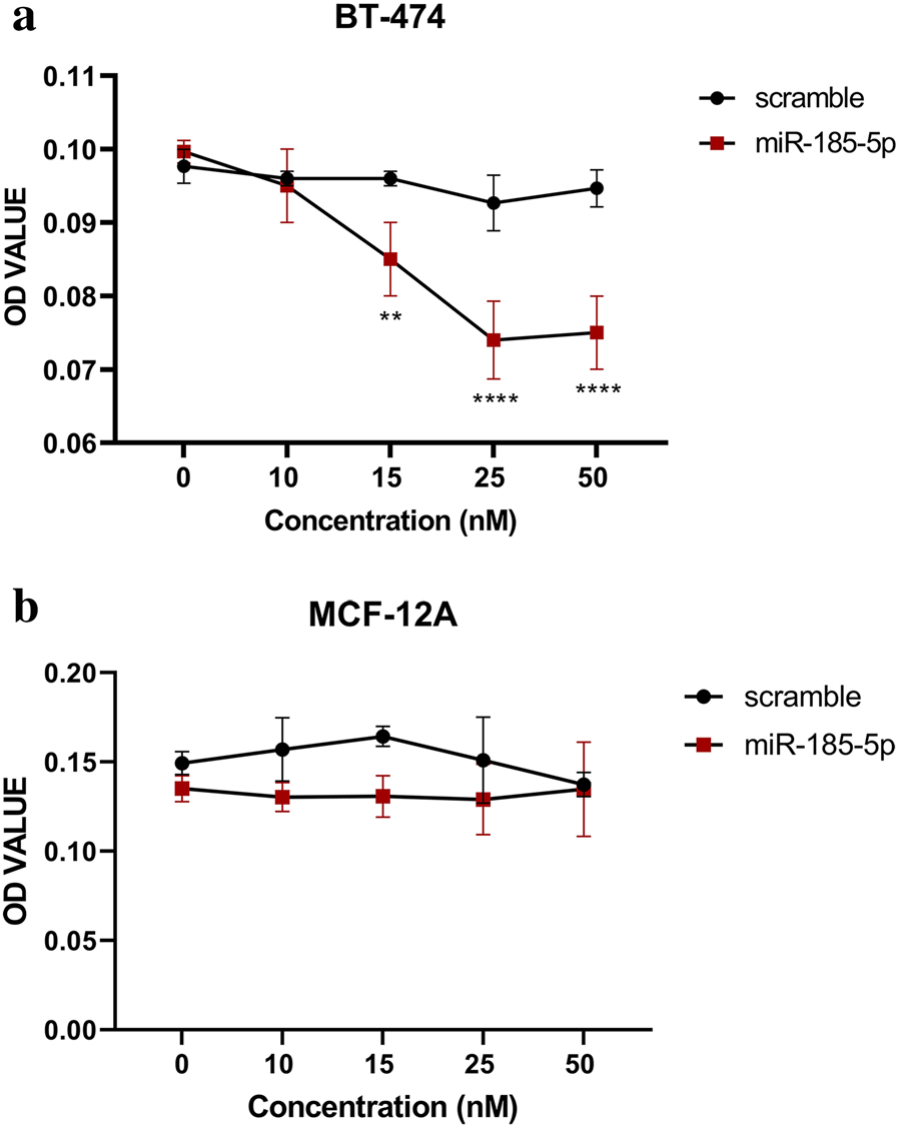
**a** The effect of miR-185-5p on BT-474 cell viability under different concentrations. The most significant inhibition of cell proliferation observed at 25 nM (**p < 0.01, ****p < 0.0001). **b** The effect of miR-185-5p on MCF-12A cell viability under different concentrations. Even under the different concentrations of miR-185-5p, no reduction in MCF-12A cell proliferation

**Fig. 2:**
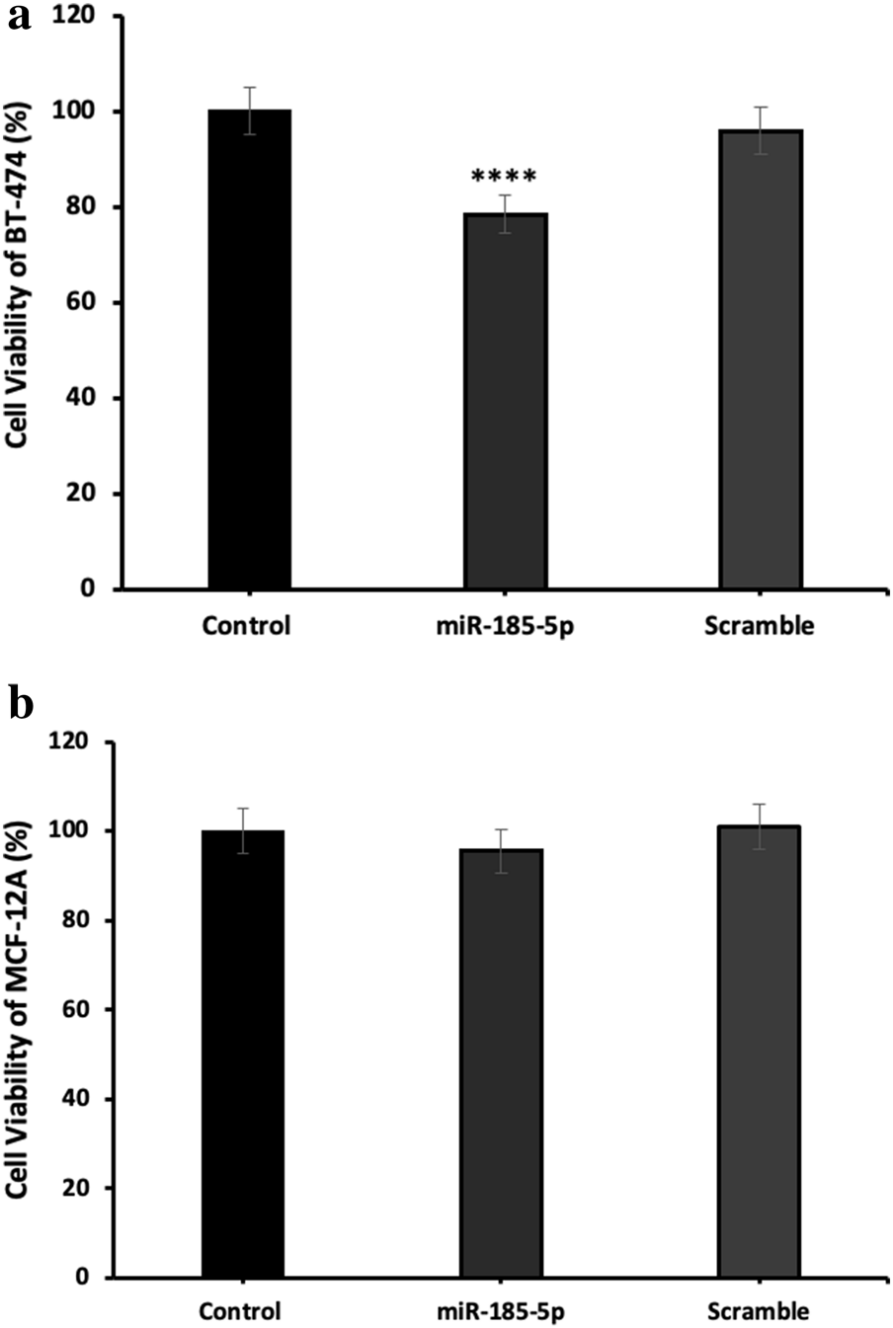
**a** miR-185-5p inhibits the BT-474 cell proliferation. The cell viability analysis performed using MTT assay by the transfection of 25 nM miR-185-5p and scramble in BT-474 cells (****p < 0.0001). **b** The overexpression of miR-185-5p in MCF-12A do not affect the cell viability significantly. MTT assay performed for the determination of the cell viability after 25 nM miR-185-5p and scramble transfection into MCF-12A cell line (Control: DMEM)

To investigate the effect of miR-185-5p on the cell proliferation of BT-474 and MCF-12A, the miR-185-5p transfection was performed in the different concentrations (0, 10, 15, 25 and 50 nM) for 24, 48 and 72 h and then MTT assay was carried out. Subsequently, the most effective concentration of miR-185-5p for BT-474 cells was determined as 25 nM at 48 h (Fig. 1a). Unlike, we observed that there was no significant reduction of cellular viability in MCF-12A under the different concentrations (Fig. 1b). 25 nM miR-185-5p was able to inhibit BT-474 cell viability by about 15% compared with the control group and scramble (p < 0.0001) (Fig. 2a). On the other hand, the ectopic expression of 25 nM miR-185-5p in the MCF-12A cells resulted in a reduction of cell viability by 3.5% compared with the control group (Fig. 2b). As a result of this data, we indicated that miR-185-5p has an anti-proliferative effect on BT-474 breast cancer cell viability.

### Transfection efficiency of miR-185-5p in BT-474

**Fig. 3:**
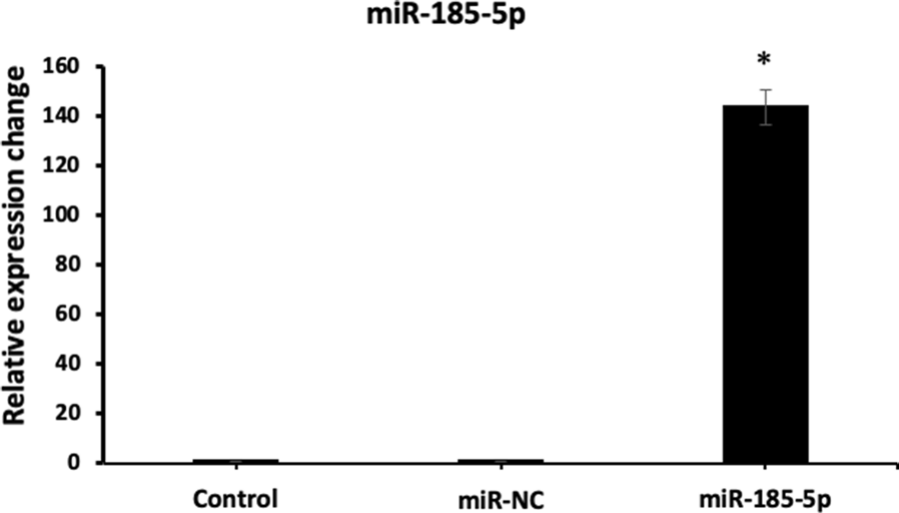
The transfection efficiency results of miR-185-5p in BT-474 cells (*p < 0.05) (Control: The level of expression given as control is proportional to the number 1, miR-NC: Scramble)

To validate the transfection efficiency of miR-185-5p in BT-474, the qRT-PCR assay was performed. The qRT-PCR data showed that miR-185-5p expression was up-regulated in breast cancer cells with transfection of miR-185-5p mimic, while there was no prominent increase in miR-185-5p levels in control group, suggesting that the ectopic expression of miR-185-5p was successfully carried out as seen Fig. 3 (p < 0.05).

### miR-185-5p induces apoptosis in breast cancer cells

**Fig. 4:**
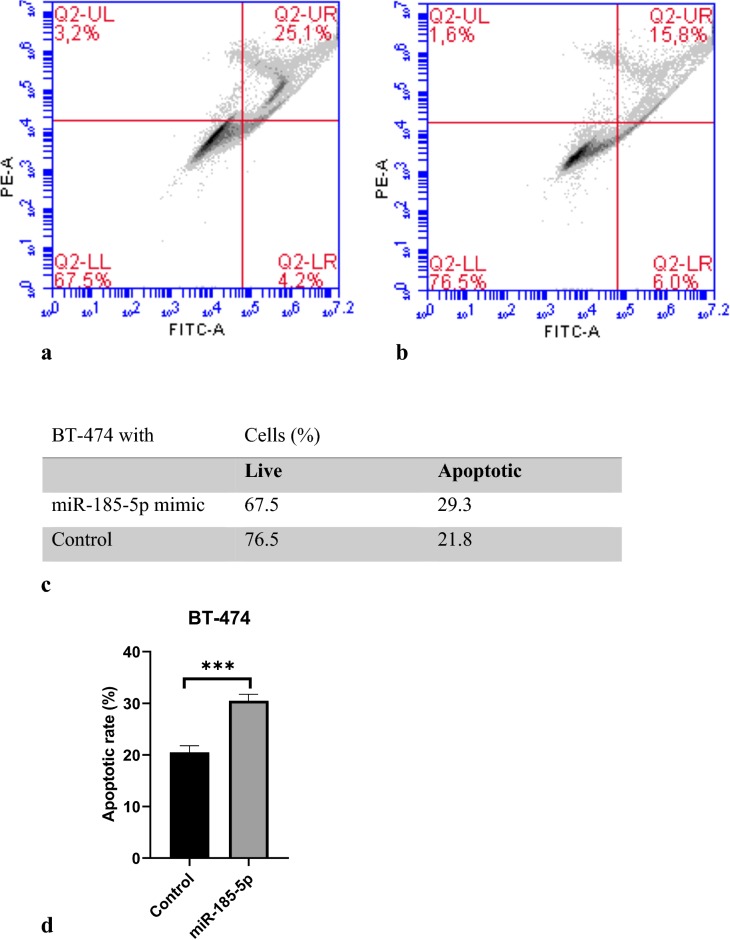
miR-185-5p effect on BT-474 cell apoptosis. **a** Flow cytometric analysis revealed miR-185-5p transfected apoptotic BT-474 cells ratio. **b** Non-transfected apoptotic BT-474 cells ratio. **c** Percentages of apoptotic and live cells. **d** Apoptotic rate of BT-474 following miRNA-185-5p transfection on graph (***p < 0.001) (Control: DMEM)

**Fig. 5:**
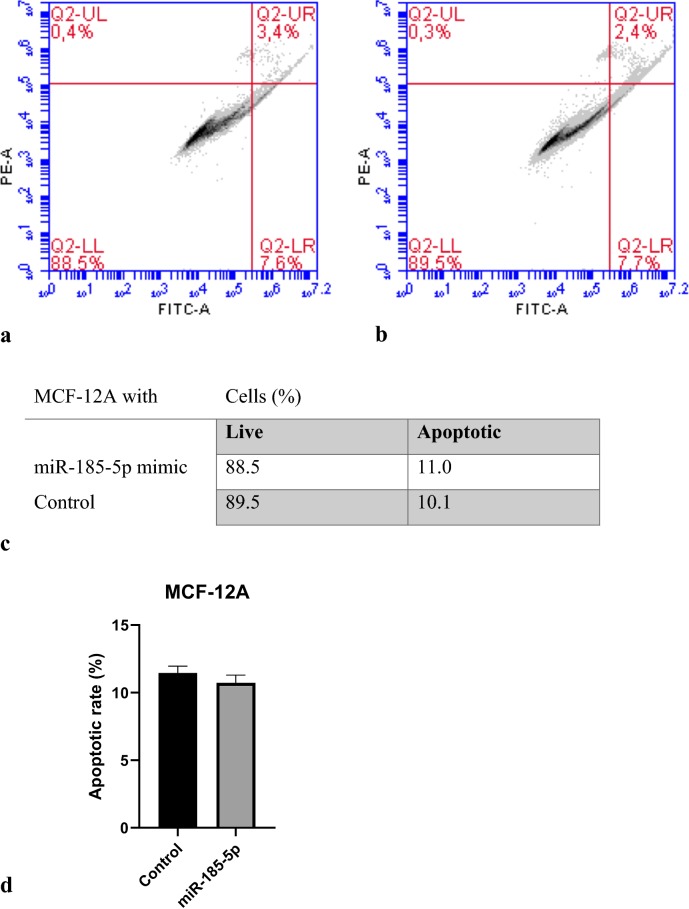
miR-185-5p effect on MCF-12A cell apoptosis. **a** Vybrant Apoptosis assay showed any significant effect of ectopic expression of miR-185-5p on cell apoptosis. **b** In control group, similar results observed with (**a**). **c** Percentages of apoptotic and live cells. **d** Apoptotic rate of MCF-12A following miRNA-185-5p transfection on graph (Control: DMEM)

To explore the mechanism by which cell viability was suppressed, the apoptosis ratio of BT-474 and MCF-12A cells transfected with miR-185-5p was measured by flow cytometry. Our data revealed that the BT-474 cell apoptosis was significantly increased with the overexpression of miR-185-5p compared to the control group. Our results suggested that miR-185-5p may be as a tumor suppressor for breast cancer (Fig. 4). In addition, the apoptosis ratio of BT-474 cells in terms of cell percentage was determined as 9% as shown in Fig. 4 (p < 0.001). In contrast, by observing the data, there was not significantly increase in the apoptosis of miR-185-5p transfected MCF-12A cells (Fig. 5). Together, these results indicated that miR-185-5p could induce the apoptosis in BT-474 breast cancer cell compared with the MCF-12A non-cancerous epithelial breast cell.

### miR-185-5p regulates the cell cycle in BT-474 cells

**Fig. 6:**
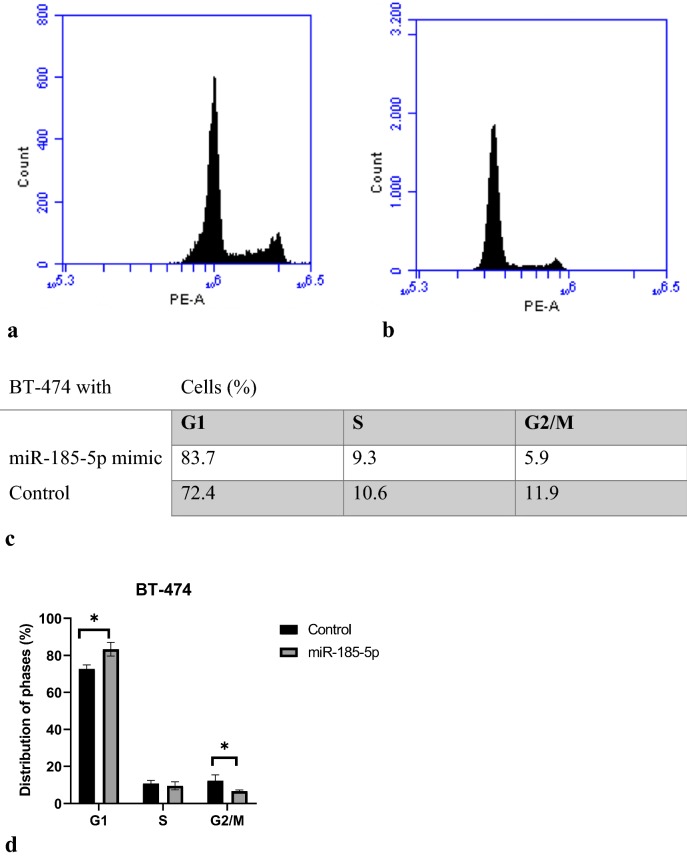
miR-185-5p regulates cell-cycle. **a** Cell- cycle analysis graph of the miR-185-5p transfected BT-474 cells. **b** Control group cell cycle analysis graph. **c** Percentages of G1, S, and G2/M phase cells. **d** Comparison of the miR-185-5p transfected and non-transfected cells cell cycle analysis results on graph (*p < 0.05) (Control: DMEM)

In order to further support the inhibitory effect of miR-185-5p on breast cancer cell proliferation, cell cycle analysis was performed by flow cytometry. The flow cytometric analysis results suggested that the proportion of cells stagnated in the G_0_/G_1_ phase was higher in the BT-474 cells transfected with miR-185-5p than the control group. Besides, we found that the number of cells transfected with the miR-185-5p in the S and G_2_/M phases was less than the control group, indicating that the miR-185-5p could block the mitosis in the breast cancer cells (Fig. 6) (p < 0.05). These results demonstrated that the inhibition of breast cancer cell viability via miR-185-5p can be mediated by regulating the cell cycle.

### miR-185-5p regulates the expression of the apoptosis-related genes

**Fig. 7:**
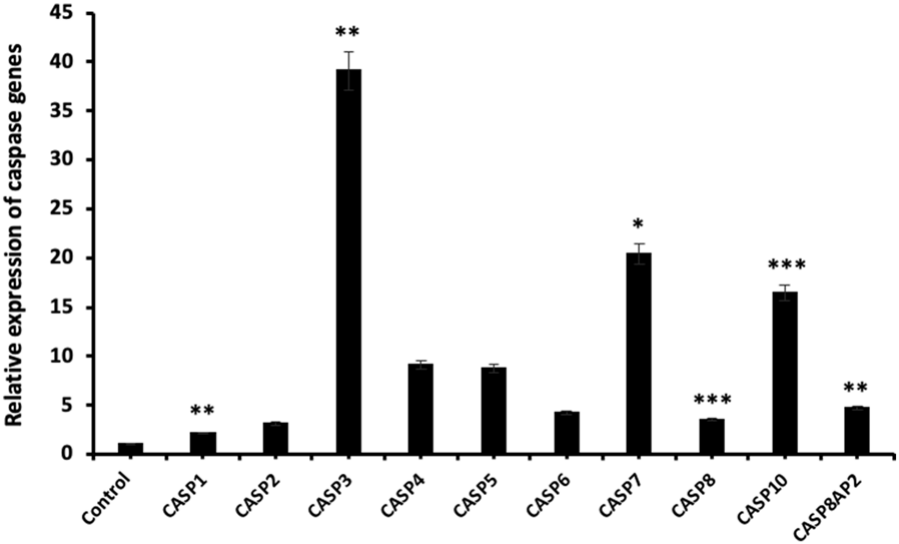
Relative expression of caspase genes after miR-185-5p transfection in BT-474 cell line (*p < 0.05, **p < 0.01, ***p < 0.001)

In order to explore the apoptotic gene expression pattern after the transfection of miRNA-185-5p in BT-474 cells, we performed real-time PCR using Human Apoptosis Primer Library assay that profiles the expression of 88 apoptosis-related genes. However, we obtained reasonable results for 69 genes by qRT-PCR (Additional file 1). According to our results, all Caspase gene family members exhibited an expression increase after transfection of miRNA-185-5p in BT-474 cell line and also the maximum increase rate was observed in Caspase 3 gene (p < 0.01) (Fig. 7).

**Fig. 8:**
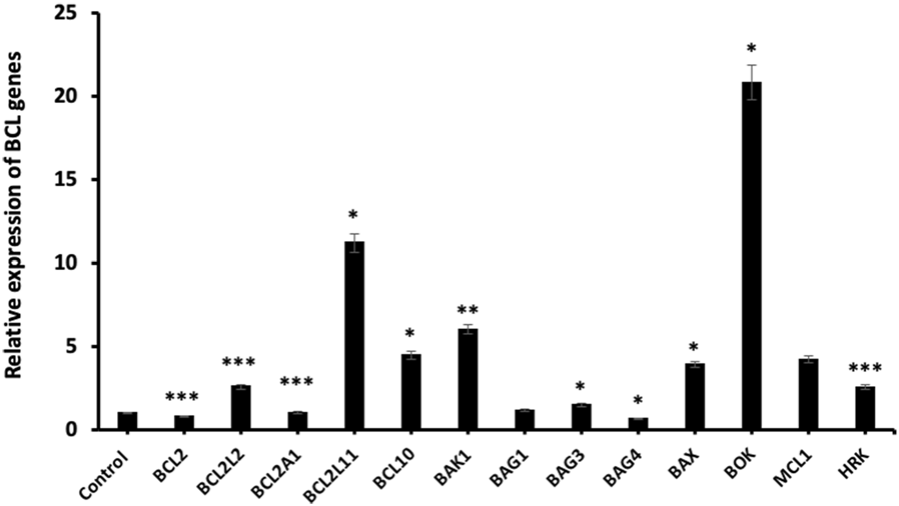
Relative expression of *BCL2* genes after miR-185-5p transfection in BT-474 cell line (*p < 0.05, **p < 0.01, ***p < 0.001)

One of the other primer group in Human Apoptosis Primer Library is *BCL2*-related gene family and we observed that the *BCL2* (p < 0.001) and *BAG4* (p < 0.05) mRNA levels were significantly decreased with miR-185-5p transfection. These results also demonstrated that *BOK* gene expression was mostly increased compared with the control group. On the other hand, there was no significant change in *BCL2A1* and *BAG1* mRNA levels (Fig. 8).

**Fig. 9:**
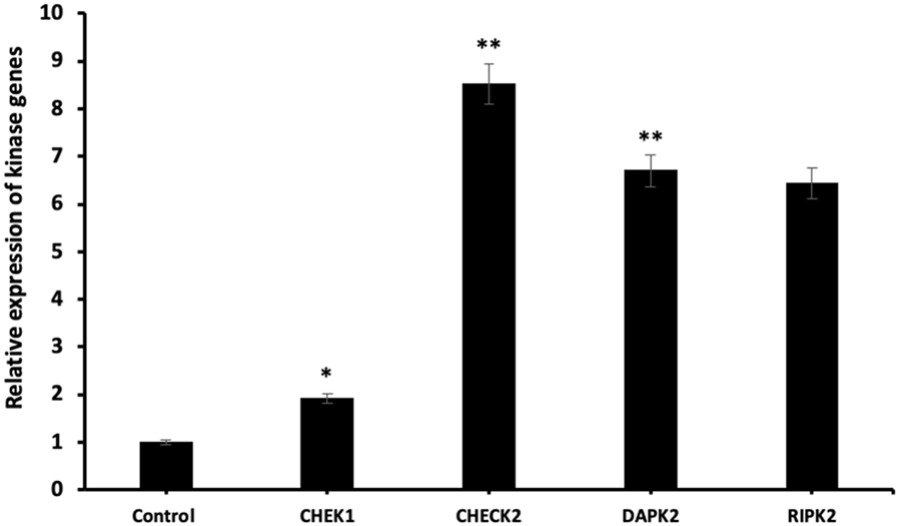
Relative expression of Kinase genes after miR-185-5p transfection in BT-474 cell line (*p < 0.05, **p < 0.01)

**Fig. 10:**
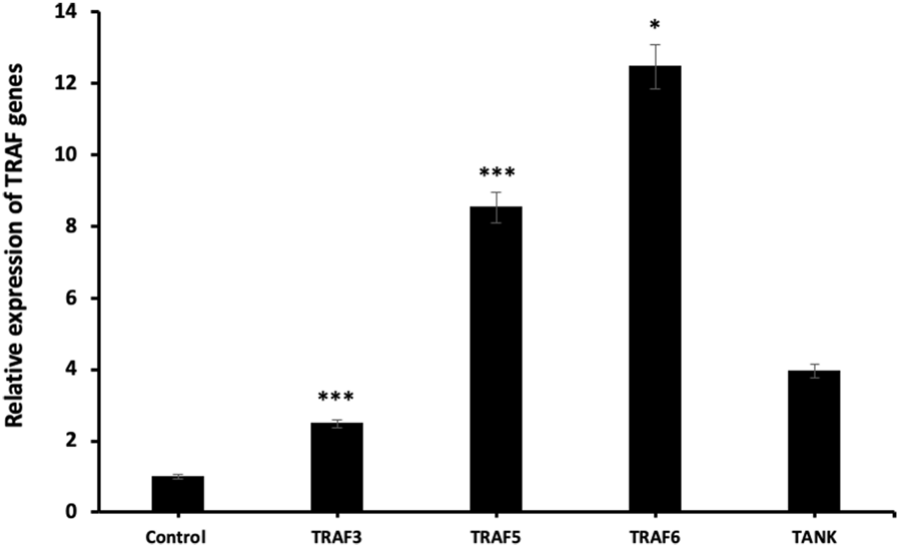
Relative expression of TRAF genes following miR-185-5p transfection in BT-474 cell line (*p < 0.05, ***p < 0.001)

We also investigated the relative expression of Kinase and TRAF gene family and following qRT-PCR results, the highest increase was observed in the mRNA expression levels of *CHECK2* (p < 0.01) and *TRAF6* (p < 0.05) (Figs. 9 and 10, respectively). In addition, the mRNA expression of the other genes was up-regulated after miR-185-5p transfection (Figs. 9, 10) (p < 0.05, p < 0.01, p < 0.001).

**Fig. 11:**
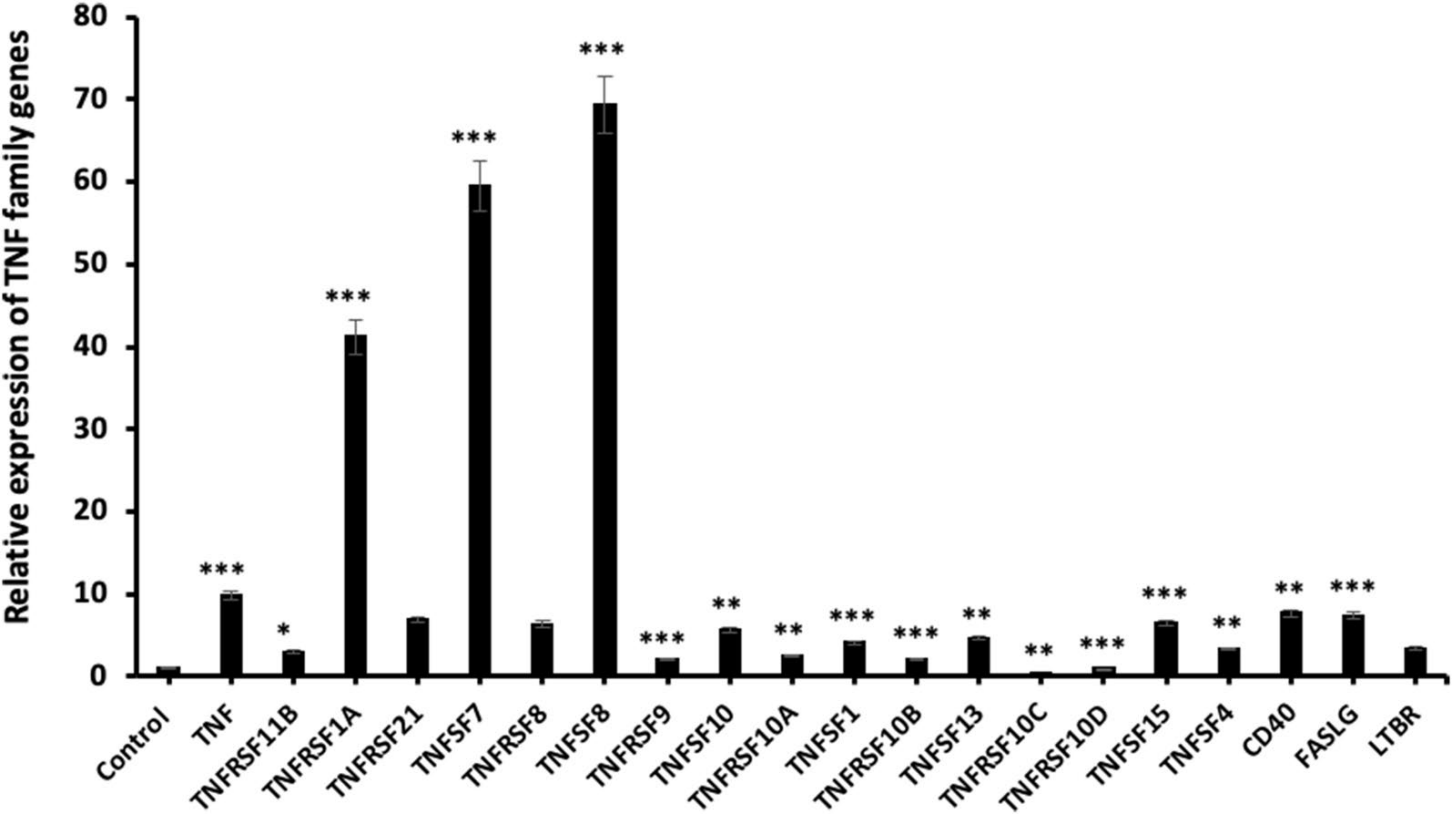
Relative expression of TNF genes with miR-185-5p transfection in BT-474 cells (*p < 0.05, ** p < 0.01, ***p < 0.001)

We also performed qRT-PCR to determine the relative expression of TNF family genes and this study revealed a substantial change in mRNA expression levels for almost all genes. *TNFRSF10C* (p < 0.01) and *TNFRSF10D* (p < 0.001) exhibited a significant increase in mRNA expression in the BT-474 cells. On the other side, the relative mRNA expression levels of the other genes were significantly increased and also *TNFSF8* (p < 0.001) was highly up-regulated in comparison with the other genes. The qRT-PCR results indicated that *TNFSF7* (p < 0.001) and *TNFRSF1A* (p < 0.001) genes mRNA levels were increased with the transfection of miR-185-5p in BT-474 cells (Fig. 11).

**Fig. 12:**
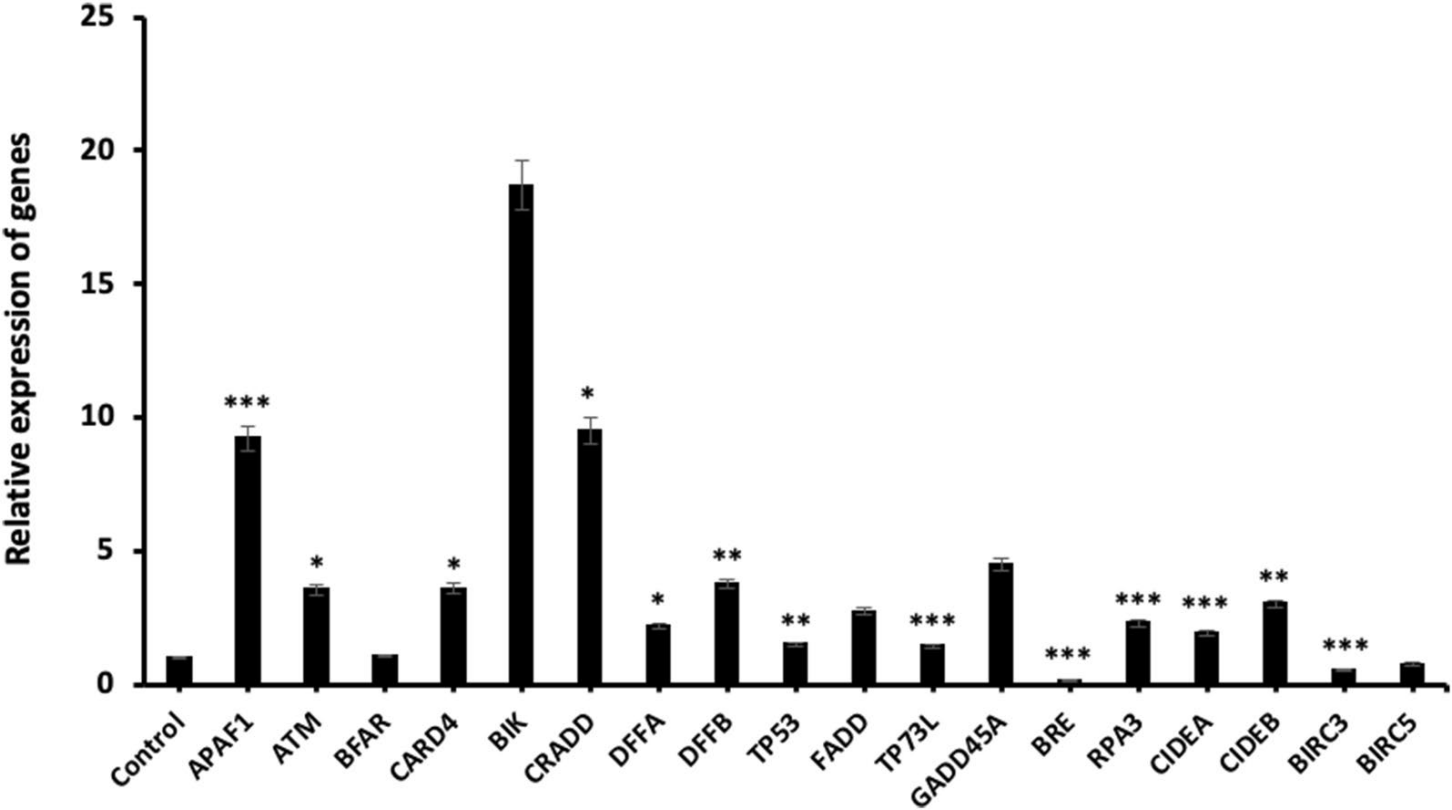
The expression analysis of apoptosis related-genes at mRNA level (*p < 0.05, **p < 0.01, ***p < 0.001)

Finally, we found that the other apoptosis-related genes groups also showed a considerable change in terms of mRNA expression levels and the highest mRNA expression increase rate was observed in *BIK* gene. According to our results, *BRE* (p < 0.001) gene expression level was decreased in miR-185-5p transfected BT-474 cells and also *BRC3* and *BRC5* genes were slightly decreased at the mRNA level. On the other hand, there was no significant change in the *BFAR* gene expression (Fig. 12). In brief, all these qRT-PCR data suggested that miR-185-5p mediated the mRNA levels of many different genes associated with apoptosis.

### miR-185-5p targets BCL2 in BT-474 breast cancer cells

**Fig. 13:**
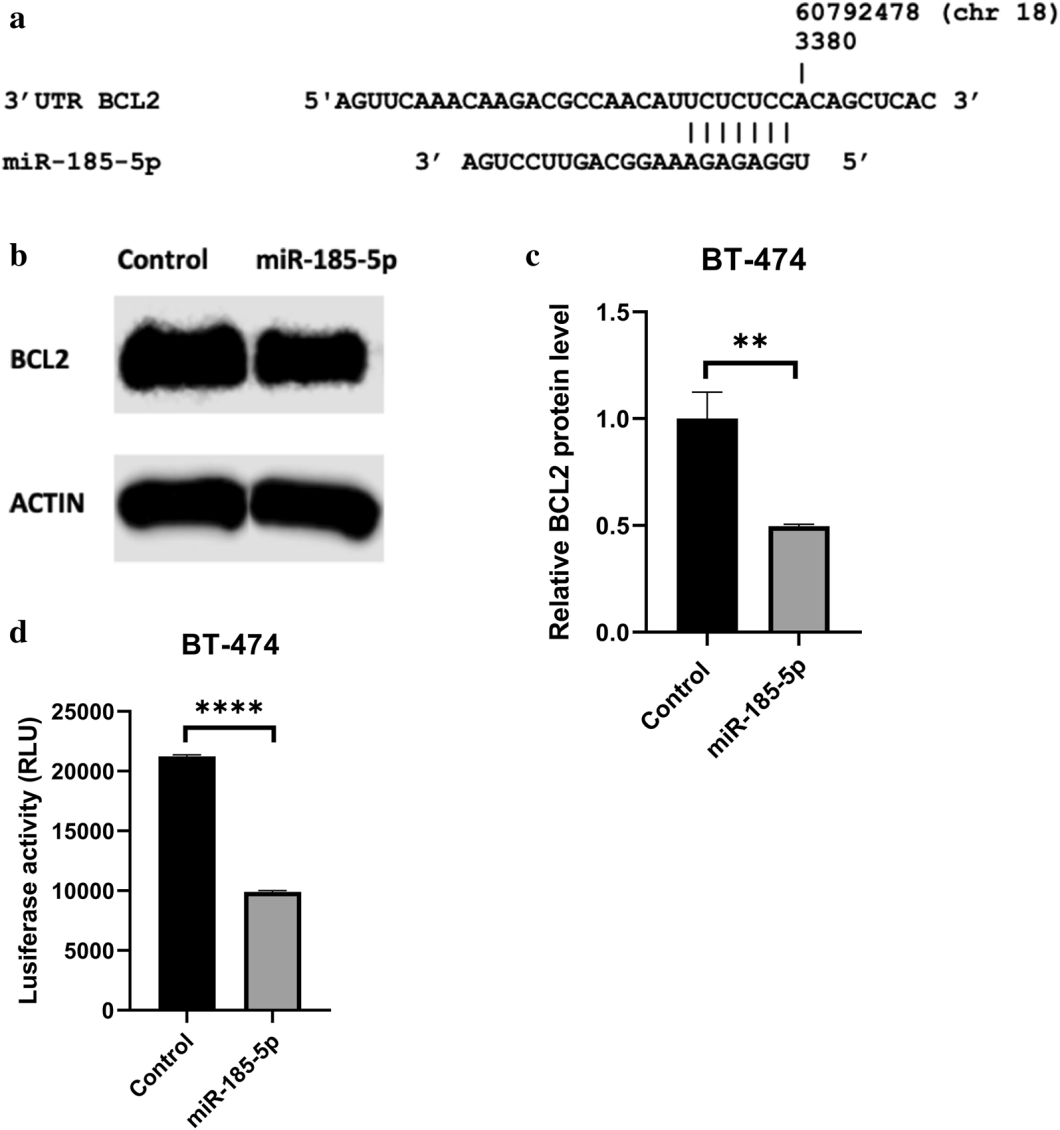
The target identification of miR-185-5p. **a** The predicted binding site between miR-185-5p and 3′UTR BCL2. **b** Western blot band results. **c** Graph of relative BCL2 protein level change (n = 3, **p < 0.01). **d** Relative luciferase activity results on graph (****p < 0.0001) (Control: DMEM)

To find out the target of miR-185-5p in breast cancer cells, firstly we identified the potential target genes of miR-185-5p by using three different miRNA target prediction databases (miRDB, DIANA, and miRSystem). BCL2, which is common in three bioinformatics tools, is selected for target analysis. Also, the potential binding site between miR-185-5p and BCL2 was predicted by miRmap database as shown in Fig. 13a. To experimentally confirm the targeting of BCL2 by miR-185-5p, BCL2 protein expression level was shown by western blot. The results indicated that the *BCL2* expression was significantly decreased after miR-185-5p transfection compared to the control group (p < 0.01) (Fig. 13b–c).

### Luciferase reporter assay

To further demonstrate whether BCL2 is a direct target of miR-185-5p, luciferase assay was performed therefore the luciferase plasmid inserted with 3′UTR of BCL2 and miR-185-5p mimic were co-transfected to BT-474 cells. These obtained data indicated that the relative luciferase activity significantly decreased with the miR-185-5p transfection compared with the control group as seen Fig. 13d (p < 0.0001). By observing the western blot and luciferase assay analysis, it was determined that miR-185-5p targeted the 3′UTR of BCL2 in the breast cancer cell.

## Discussion

Although breast cancer can be diagnosed by improved diagnostic techniques and cured with different therapeutic approaches, this cancer is still the second leading cause of cancer-related death in woman worldwide [23, 24]. Some complications such as high recurrence rate and the formation of resistance to chemotherapy and endocrine therapy obstruct to combat breast cancer [25, 26].

Investigating the underlying molecular mechanisms of breast cancer at great length and the development of new therapeutic approaches are needed to provide better treatment for breast cancer patients. One of the well-known naturally occurring compounds is usnic acid which is a type of most commonly studied lichen secondary metabolite and it exerts an anti-proliferative effect on many cancer cell lines [6, 27] such as glioma cells (PRCC and U87MG) [28], human gastric cells (BGC823, SGC7901 and AGS) [29, 30], human lung carcinoma (A549) [8], prostate cancer cell (CWR22Rv-1) [30], human colon adenocarcinoma cell (HT-29) [30], and breast cancer cells (MCF-7, BT-474 and MDA-MB-231) [22]. Besides anti-proliferative properties, usnic acid can also inhibit tumor progression via anti-angiogenesis and anti-invasion activity through manipulating different signaling pathways in different cancer cells, including lung cancer cells (A549, H1650 and H1975) [31] and breast cancer cells (Bcap-37) [32].

Accompanied by the discovery of miRNAs and their investigations at the molecular level in cancer biology, it was observed that miRNAs have been deregulated in many human cancer cells [33–35]. Moreover, several studies have demonstrated that the miRNAs can act as a key regulator for oncogenes or tumor suppressor genes in cancer development and progression by mediating vital cellular processes including cell proliferation, differentiation, and apoptosis [36]. Due to the main cellular role of miRNAs in cancer progression, miRNAs gained attention as a novel therapeutic approach for cancer treatment. Indeed, many studies revealed that tumor development can be managed by miRNAs by inhibition of pro-oncogenic miRNAs or re-introduction of tumor suppressor miRNAs into breast cancer cells [37]. Han et al. [38] showed that the miR-1307-3p stimulates breast cancer development by targeting SMYD4, while downregulation of miR-1307-3p could inhibit the cell proliferation in breast cancer cells. Zou et al. [39] demonstrated that overexpression of the miRNA-375 could inhibit the cell viability, invasion and migration by targeting PAX6. Moreover, Ji et al. [40] indicated that the miR-3196 was significantly downregulated in breast cancer tissues compared with adjacent normal tissues and ectopic expression of miR-3196 in breast cancer cells could suppress cell proliferation via inducing apoptosis by targeting ERBB3. Tang et al. investigated the effect of miR-185 on tumor progression in various breast cancer cell lines. As a result of this study, it was demonstrated that miR-185 expression decreased in BT-474 breast cancer cell [41]. Ostadrahimi et al. [42] revealed that the miR-185-5p can trigger apoptosis in 12.82% and 9.2% of DU145 and PC3 prostate cancer cell population, respectively. Our previous study demonstrated that miR-185-5p, the most promising candidate usnic acid response miRNA, was remarkably up-regulated through usnic acid treatment to BT-474 breast cancer cell [22]. In this study, we tested that transfection of miR-185-5p in BT-474 breast cancer cell might suppress tumorigenicity by inhibiting cell proliferation and promoting apoptosis. In addition, we focused on the function of the miR-185-5p in breast cancer cells thus the underlying molecular mechanisms mediated by miR-185-5p on cell apoptosis was investigated in BT-474 cells.

To elucidate the role of miR-185-5p on cell proliferation and apoptosis, MTT assay and flow cytometry analysis were conducted in BT-474 cells and the results showed that the miR-185-5p decreased cell viability. Our cell viability and apoptosis assay results revealed that there is no significant effect of miR-185-5p on cell viability of normal breast cells (MCF-12A), suggesting that miR-185-5p can inhibit breast cancer cell proliferation without detriment to normal breast cells. Hao et al. [43] showed that cell apoptosis rate was increased under the miR-374c-5p transfection by about 12% and 14% in BT-549 and MDA-MB-231 breast cancer cell lines, respectively. In other study, Cui et al. [43] demonstrated that tumor progression was suppressed by miR-216a, stimulating cell apoptosis by 27% in MCF-7 breast cancer cell line. Qin et al. also reported that miR-99a-5p increased apoptosis rate 15% in MCF-7 and MDA-MB-231 breast cancer cells. Our data showed that the miR-185-5p obviously induced cell apoptosis in the 9% ratio, whereas there was no significant change in apoptosis ratio in MCF-12A normal breast cancer cells.

Apoptosis is a programmed cell death process that provides homeostasis in tissues and mediated by extrinsic and intrinsic pathway. Several in vitro studies have documented a role for miRNAs in apoptosis of human cancer cells [44, 45]. In the literature, there are many studies performed with flow cytometry and western blot assay to determine the apoptosis rate of miRNAs. Therefore, there are few studies that determine the mRNA level in relation to apoptosis of miRNAs. In our study, apoptosis data obtained after flow cytometry assay was analyzed by qRT-PCR technology in mRNA level. Liu et al. [44] reported western blot assay and real-time PCR were used to measure apoptosis related factors Bax and BCL-2 expression level. Liu et al. studies have shown that Bax expression level significantly decreased, but BCL-2 expression significantly increased compared with the control. In another studies, Li et al. showed that miR-886-5p negatively regulates Bax up-regulation of miR-886-5p in cancer cell line. This study revealed the role of miR-886 in apoptosis in cervical squamous cell carcinoma by using qRT-PCR and western blot assays [46]. The gene expression levels of our study showed similar levels with the literature. BCL2 is a well-known regulator gene which inhibits apoptosis by contributing the intrinsic apoptosis pathway [47, 48]. In more recent studies, it has been reported that tumorigenesis could be abolished by inducing cell apoptosis via miRNA-mediated BCL2 downregulation in different cancer cells. Yu et al. [49] demonstrated that apoptosis can be induced via directly targeting the 3′UTR of BCL2 by miRNA-136 in human gastric cancer cells. Zhu et al. [50] elucidated that miR-365 targeted the BCL2 thus and could suppress cell growth by promoting apoptosis in melanoma cells. Li et al. [51] revealed that miR-34c involved in the regulation of cell apoptosis by targeting the 3′UTR of BCL2, which led to inhibition of the cell viability in M4e laryngeal carcinoma cells. Pekarsky et al. indicated that the expression of miR-15/16 do not observe in MEG-01 leukemic cells, whereas the ectopic expression of miR-15/16 promoted cell apoptosis via direct targeting of BCL2 in leukemic cells [52]. In this study, we also demonstrated that miR-185-5p targeted the BCL2 in BT-474 cells, therefore, miR-185-5p significantly increased the cell apoptosis, suggesting that miR-185-5p can act as a tumor suppressor in breast cancer cells. This is the first study based on the determination of the expression level of all genes in the apoptosis pathway by miR-185-5p transfection.

The possible candidate gene of miR-185-5p was identified by bioinformatic miRNA target prediction tools, which showed that the 3′UTR of BCL2 has a binding site with miR-185-5p seed region. Luciferase reporter assay results showed that the increased expression of miR-185-5p in BT-474 cells inhibited the luciferase activity of reporter plasmid with 3′UTR of BCL2, confirming that the miR-185-5p could negatively regulate the BCL2 expression in breast cancer cells. To the best our knowledge, our results firstly uncovered that BCL2 is a target of miR-185-5p in BT-474 cells. Li et al. [53] suggested that the miR-148a directly targeted BCL2 by binding with 3′UTR sequence of BCL2 mRNA in MCF-7 breast cancer cell line.

## Conclusion

In conclusion, we investigated the potential underlying mechanisms of miR-185-5p on apoptosis in BT-474 cells. Our findings showed that the up-regulation of miR-185-5p could block the cell growth by stimulating to apoptosis through directly targeting the 3′UTR of BCL2. It was suggested that usnic acid-induced BCL2 downregulation might be regulated by miR-185-5p in BT-474 breast cancer cell line. Therefore miR-185-5p could be considered as a tumor suppressor in BT-474 cells and might serve as a potential therapeutic candidate in breast cancer treatment. Further studies involving in vivo and clinical studies need to confirm the therapeutic effect of miR-185-5p on breast cancer.

## Supplementary information

**Additional file 1.** The relative gene expression analysis of apoptosis related genes.

## Data Availability

The datasets used and/or analyzed in the current study are available from the corresponding author on reasonable request.

## Abbreviations

3′ UTR: 3′ untranslated region
APAF1: Apoptotic protease activating factor
ATCC: American Type culture collection
ATM: Ataxia telangiectasia mutated
BAG1: BCL2-associated athanogene
BAG3: BCL2-associated athanogene 3
BAG4: BCL2-associated athanogene 4
BAK1: BCL2-antagonist/killer 1
BAX: BCL2-associated X protein
BCL10: B-cell CLL/lymphoma 10
BCL2: B-cell CLL/lymphoma 2
BCL2A1: BCL2-related protein A1
BCL2L11: BCL2-like 11 (apoptosis facilitator)
BCL2L2: BCL2-like 2
BFAR: Bifunctional apoptosis regulator
BIK: BCL2-interacting killer (apoptosis-inducing)
BIRC3: Baculoviral IAP repeat-containing 3
BIRC5: Baculoviral IAP repeat-containing 5 (survivin)
BOK: BCL2-related ovarian killer
BRE: Brain and reproductive organ-expressed
CARD4: Caspase recruitment domain family, member 4
CASP1: Caspase 1, apoptosis-related cysteine protease
CASP10: Caspase 10, apoptosis-related cysteine protease
CASP2: Caspase 2, apoptosis-related cysteine protease
CASP3: Caspase 3, apoptosis-related cysteine protease
CASP4: Caspase 4, apoptosis-related cysteine peptidase
CASP5: Caspase 5, apoptosis-related cysteine protease
CASP6: Caspase 6, apoptosis-related cysteine protease
CASP7: Caspase 7, apoptosis-related cysteine protease
CASP8: Caspase 8, apoptosis-related cysteine protease
CASP8AP2: CASP8 associated protein 2
CD40: CD40 antigen (TNF receptor superfamily member 5)
CHEK1: CHK1 checkpoint homolog (*S. pombe*)
CHEK2: CHK2 checkpoint homolog (*S. pombe*)
CIDEA: Cell death-inducing DFFA-like effector a
CIDEB: Cell death-inducing DFFA-like effector b
CRADD: CASP2 and RIPK1 domain containing adaptor
DAPK2: Death-associated protein kinase 2
DDT: Dichlorodiphenyltrichloroethane
DFFA: DNA fragmentation factor, 45 kDa, alpha polypeptide
DFFB: DNA fragmentation factor, 40 kDa, beta polypeptide
DMEM: Dulbecco’s modified Eagle’s medium
DMSO: Dimethyl sulfoxide
DNA: Deoxyribonucleic acid
EGF: Epidermal Growth Factor
FADD: Fas (TNFRSF6)-associated via death domain
FASLG: Fas ligand (TNF superfamily, member 6)
FBS: Fetal Bovine Serum
G1 Phase: Gap1 Phase
G2 Phase: Gap2 phase
GADD45A: Growth arrest and DNA-damage-inducible, alpha
GAPDH: Glyceraldehyde-3-phosphate dehydrogenase
HRK: Harakiri, BCL2 interacting protein
HRP: Horseradish peroxide
LTBR: Lymphotoxin beta receptor (TNFR superfamily, member 3)
M Phase: Mitotic Phase
MAPK: Mitogen-activated protein kinase
MCL1: Myeloid cell leukemia sequence 1 (BCL2-related)
miR-185-5p: microRNA-185-5p
miRNA: microRNA
MTT: 3-(4,5-Dimethylthiazol-2-yl)-2,5-diphenyltetrazolium bromide
NM: Nano molar
PBS: Phosphate buffered saline
PI: Propidium iodide
qRT-PCR: Quantitative reverse transcription polymerase chain reaction
RIPK2: Receptor-interacting serine-threonine kinase 2
RPA3: Replication protein A3, 14 kDa
S Phase: Synthesis phase
SDS-PAGE: Sodium dodecyl sulfate-polyacrylamide
SEM: Standard error of the mean
TANK: TRAF family member-associated NFKB activator
TBST: Tris-Buffered Saline Tween
TGF BETA: Transforming growth factor beta
TNF: Tumor necrosis factor (TNF superfamily, member 2)
TNFRSF10A: Tumor necrosis factor receptor superfamily, member 10a
TNFRSF10B: Tumor necrosis factor receptor superfamily, member 10b
TNFRSF10C: Tumor necrosis factor receptor superfamily, member 10c
TNFRSF10D: Tumor necrosis factor receptor superfamily, member 10d
TNFRSF11B: Tumor necrosis factor receptor superfamily, member 11b
TNFRSF1A: Tumor necrosis factor receptor superfamily, member 1A
TNFRSF21: Tumor necrosis factor receptor superfamily, member 21
TNFRSF8: Tumor necrosis factor receptor superfamily, member 8
TNFRSF9: Tumor necrosis factor receptor superfamily, member 9
TNFSF10: Tumor necrosis factor (ligand) superfamily, member 10
TNFSF13: Tumor necrosis factor (ligand) superfamily, member 12
TNFSF15: Tumor necrosis factor (ligand) superfamily, member 15
TNFSF4: Tumor necrosis factor (ligand) superfamily, member 4
TNFSF7: Tumor necrosis factor (ligand) superfamily, member 7
TNFSF8: Tumor necrosis factor (ligand) superfamily, member 8
TP53: Tumor protein p53 (Li-Fraumeni syndrome)
TP73L: Tumor protein p73-like
TRAF3: TNF receptor-associated factor 3
TRAF5: TNF receptor-associated factor 5
TRAF6: TNF receptor-associated factor 6
