# Supplementary material for: miR-185-5p response to usnic acid suppresses proliferation and regulating apoptosis in breast cancer cell by targeting Bcl2
Source: Biol Res. 2020 May 4;53:19. doi: 10.1186/s40659-020-00285-4 (PMC7197166; doi:10.1186/s40659-020-00285-4)
Supplement: Supplementary file 1 — Additional file 1. The relative gene expression analysis of apoptosis related genes. [file 40659_2020_285_MOESM1_ESM.docx]

**Additional Data**

**Table S1. The relative gene expression analysis of apoptosis related genes**

| **Gene Family** | **Gene** | **Expression Fold Change** | **p-value** |
| --- | --- | --- | --- |
| **Caspase Family** | *CASP1* | 2,14 | p<0.01 |
|  | *CASP2* | 3,11 | - |
|  | *CASP3* | 39,12 | p<0.01 |
|  | *CASP4* | 9,16 | - |
|  | *CASP5* | 8,78 | - |
|  | *CASP6* | 4,23 | - |
|  | *CASP7* | 20,39 | p<0.05 |
|  | *CASP8* | 3,56 | p<0.001 |
|  | *CASP10* | 16,51 | p<0.001 |
|  | *CASP8AP2* | 4,72 | p<0.01 |
| **Kinase Family** | *CHEK1* | 1,92 | p<0.05 |
|  | *CHECK2* | 8,51 | p<0.01 |
|  | *DAPK2* | 6,70 | p<0.01 |
|  | *RIPK2* | 6,43 | - |
| **BCL2 Family** | *BCL2* | 0,78 | p<0.001 |
|  | *BCL2L2* | 2,58 | p<0.001 |
|  | *BCL2A1* | 1,04 | p<0.001 |
|  | *BCL2L11* | 11,20 | p<0.05 |
|  | *BCL10* | 4,50 | p<0.05 |
|  | *BAK1* | 6,04 | p<0.01 |
|  | *BAG1* | 1,17 | - |
|  | *BAG3* | 1,50 | p<0.05 |
|  | *BAG4* | 0,65 | p<0.05 |
|  | *BAX* | 3,93 | p<0.05 |
|  | *BOK* | 20,82 | p<0.05 |
|  | *MCL1* | 4,21 | - |
|  | *HRK* | 2,57 | p<0.001 |
| **TRAF Family** | *TRAF3* | 2,48 | p<0.001 |
|  | *TRAF5* | 8,51 | p<0.001 |
|  | *TRAF6* | 12,47 | p<0.05 |
|  | *TANK* | 3,96 | - |
| **TNF Family** | *TNF* | 9,78 | p<0.001 |
|  | *TNFRSF11B* | 3,00 | p<0.05 |
|  | *TNFRSF1A* | 41,21 | p<0.001 |
|  | *TNFRSF21* | 6,96 | - |
|  | *TNFSF7* | 59,51 | p<0.001 |
|  | *TNFRSF8* | 6,39 | - |
|  | *TNFSF8* | 69,31 | p<0.001 |
|  | *TNFRSF9* | 2,01 | p<0.001 |
|  | *TNFSF10* | 5,66 | p<0.01 |
|  | *TNFRSF10A* | 2,53 | p<0.01 |
|  | *TNFSF1* | 4,13 | p<0.001 |
|  | *TNFRSF10B* | 2,11 | p<0.001 |
|  | *TNFSF13* | 4,64 | p<0.01 |
|  | *TNFRSF10C* | 0,36 | p<0.01 |
|  | *TNFRSF10D* | 0,98 | p<0.001 |
|  | *TNFSF15* | 6,48 | p<0.001 |
|  | *TNFSF4* | 3,33 | p<0.01 |
|  | *CD40* | 7,70 | p<0.001 |
|  | *FASLG* | 7,39 | p<0.01 |
|  | *LTBR* | 3,46 | - |
| **Different Genes** | *APAF1* | 9,22 | p<0.001 |
|  | *ATM* | 3,54 | p<0.05 |
|  | *BFAR* | 1,05 | - |
|  | *CARD4* | 3,59 | p<0.05 |
|  | *BIK* | 18,70 | - |
|  | *CRADD* | 9,51 | p<0.05 |
|  | *DFFA* | 2,19 | p<0.05 |
|  | *DFFB* | 3,76 | p<0.01 |
|  | *TP53* | 1,51 | p<0.01 |
|  | *FADD* | 2,73 | - |
|  | *TP73L* | 1,43 | p<0.001 |
|  | *GADD45A* | 4,48 | - |
|  | *BRE* | 0,14 | p<0.001 |
|  | *RPA3* | 2,30 | p<0.001 |
|  | *CIDEA* | 1,94 | p<0.001 |
|  | *CIDEB* | 3,02 | p<0.01 |
|  | *BIRC3* | 0,52 | p<0.001 |
|  | *BIRC5* | 0,76 | - |
